# The Role of PEDF in Reproductive Aging of the Ovary †

**DOI:** 10.3390/ijms231810359

**Published:** 2022-09-08

**Authors:** Luba Nemerovsky, Hadas Bar-Joseph, Anat Eldar-Boock, Rana Tarabeih, Cindy Elmechaly, Ido Ben-Ami, Ruth Shalgi

**Affiliations:** 1 Department of Cell and Developmental Biology, Sackler Faculty of Medicine, Tel-Aviv University, Ramat-Aviv, Tel-Aviv 69978, Israel; 2 The TMCR Unit, Sackler Faculty of Medicine, Tel-Aviv University, Ramat-Aviv, Tel-Aviv 69978, Israel; 3 IVF and Infertility Unit, Department of Obstetrics and Gynecology, Shaare Zedek Medical Center, The Hebrew University Medical School of Jerusalem, Jerusalem 9103102, Israel

**Keywords:** PEDF, reproductive aging, oocyte maturation, granulosa cells

## Abstract

Reproductive aging is characterized by a decline in ovarian function and in oocytes’ quantity and quality. Pigment epithelium-derived factor (PEDF), a pivotal player in ovarian angiogenic and oxidative balance, was evaluated for its involvement in reproductive aging. Our work examines the initial stage of reproductive aging in women and mice, and the involvement of PEDF in the process. Granulosa cells from reproductively-aged (RA) women and mice (36–44 years old and 9–10 months old, respectively) indicated an increase in the level of PEDF mRNA (qPCR), with yet unchanged levels of AMH and FSHR mRNAs. However, the PEDF protein level in individual women showed an intra-cellular decrease (ELISA), along with a decrease in the corresponding follicular fluid, which reflects the secreted fraction of the protein. The in vitro maturation (IVM) rate in the oocytes of RA mice was lower compared with the oocytes of young mice, demonstrated by a reduced polar body extrusion (PBE) rate. The supplementation of PEDF improved the hampered PBE rate, manifested by a higher number of energetically-competent oocytes (ATP concentration and mtDNA copy number of individual oocytes). Our findings propose PEDF as an early marker of reproductive aging, and a possible therapeutic in vitro agent that could enhance the number of good-quality oocytes in older IVF patients.

## 1. Introduction

Reproductive aging is a process that begins in women at their mid-to-late 30s, characterized by a gradual decline in ovarian function, the number of estrogen-secreting growing follicles [1], and oocytes’ quantity and quality [2]. In general, the decline in the number of growing follicles is accompanied by disruption of the hormonal feedback [3] and narrowing of the fertility window until its final shut down. This process, which continues for several years, can be categorized into three phases [4]: (i) the initial stage of ovarian aging, in which the hormonal feedback axis changes to compensate for the reduced ovarian activity, the level of follicular-stimulating hormone (FSH) increases, and anti-Müllerian hormone (AMH) decreases; (ii) progressive ovarian aging, during which, the ongoing follicle loss leads to irregular cycles, the irregularity of follicle development and estradiol levels [5,6], and to a dramatic increase of systemic FSH level; (iii) the final menstrual period, in which only low ovarian hormone secretion can be detected [4].

Reproductive aging and fertility decrease are associated with various physiologic and molecular changes. Changes were detected in both follicular components: the oocyte and granulosa cells (GCs), which maintain an essential bi-directional relationship needed for the ovulation of a good-quality oocyte [7]. Reports point to excessive aneuploidy in the oocytes of reproductively-aged (RA) patients, manifested by errors in chromosome number [8], and mitochondrial dysfunction, due to over-production of reactive oxygen species (ROS; [9]). Hampered mitochondrial function may obstruct the oocyte’s proper maturation performance [10,11], reflected by a decreased germinal vesicle (GV) breakdown (GVBD) and first polar-body extrusion (PBE) [12,13]. Moreover, changes of mRNA, proteome, and transcriptome profiles were reported in the primary GCs (pGCs) of RA women (≥35 y; [14]) and mice. Ye et al. reported 371 differentially expressed genes between ovarian secondary follicles of RA mice (32 weeks old) compared to those of young mice (9 weeks old; [15]). These genes belong to various clusters of diverse biological processes associated with reproductive aging, such as immune response, DNA transcription and replication, and apoptosis.

Pigment epithelium-derived factor (PEDF) is a 50-kDa secreted glycoprotein [16] that is highly expressed in the female reproductive system. We have previously characterized the role of PEDF in the ovary, and have shown that the GCs of both rodents and humans produce and secrete PEDF in a hormonally-dependent manner, inversely to the upregulation of vascular endothelial growth factor (VEGF); estradiol, luteinizing hormone (LH), and progesterone downregulate the GCs’ PEDF expression and secretion [17,18]. Moreover, our findings demonstrate its anti-angiogenic, anti-inflammatory [19], and anti-oxidative [20,21] traits, and suggest that several pathologies, such as ovarian hyper-stimulation syndrome (OHSS; [17,19]) and polycystic ovary syndrome (PCOS; [22]), are associated with a low endogenic ovarian level of PEDF. Our latest study points to the ability of PEDF to attenuate oxidative stress (OS) in oocytes, reflected by improved GVBD and PBE rates, as well as by ATP concentration and mitochondrial DNA (mtDNA) copy number [21]. Our work, so far, places PEDF as a central factor in the ovary, and an attractive therapeutic agent.

PEDF mRNA expression was found to be altered in age-related diseases, such as macular degeneration, Alzheimer’s disease, and Parkinson’s disease [23], and its serum concentration reduces with age [24]. However, little is known regarding the role of PEDF in ovarian aging. As many efforts are invested in preventing and overcoming the reduced fertility of patients in their 40s, our aim was to assess PEDF involvement in the onset of reproductive aging.

Our work examines the initial stage of reproductive aging in women and mice, and the involvement of PEDF in the process. Our findings propose PEDF as an early marker of reproductive aging and a possible therapeutic in vitro agent that could enhance the number of good-quality oocytes in older IVF patients. Additional tests will be required to establish a calibrated, clinically-used tool.

## 2. Results

### 2.1. Expression of PEDF mRNA in Mice Primary Granulosa Cells (mpGCs) of RA Mice

**Figure 1:**
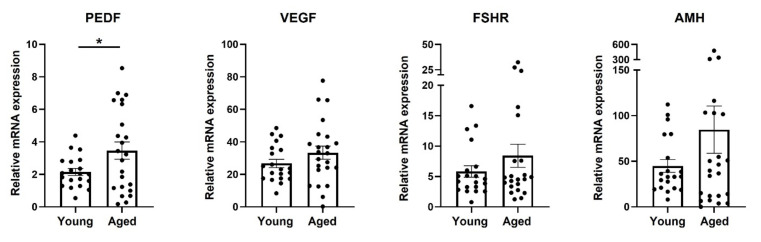
mRNA expression of selected genes in mouse granulosa cells. Mouse granulosa cells were isolated from individual young (“Young”; 6–8 weeks old) and RA (“Aged”; 40–46 weeks old) ICR female mice, primed for 48 h with PMSG (5IU or 7.5 IU, respectively). Cells were then subjected to qPCR analysis of PEDF, VEGF, FSHR, and AMH expression. Number of mice in each group: n > 15; each black circle represents one mouse. Data are presented as mean ± SEM. Results were analyzed by Student’s *t*-test with Welch’s correction, * pv ˂ 0.05.

We followed the level of mRNA expression of selected genes that are associated with fertility and reproductive aging, in the mpGCs of young and RA mice. We detected a significant increase in the expression level of PEDF mRNA in the GCs of RA mice compared to young mice (approximately 1.5-fold change). Interestingly, in the GCs of the two age groups, we detected similar mRNA levels of VEGF, AMH, and FSHR (Figure 1), as well as of the three PEDF receptors; namely, LR, ATGL, and LRP6.

### 2.2. Expression of PEDF mRNA in hpGCs of IVF Patients

**Figure 2:**
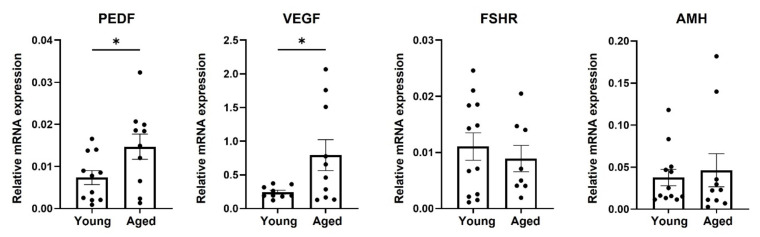
mRNA expression of selected genes in hpGCs. Human primary granulosa cells were isolated from follicular fluids of two groups of IVF patients: (1) young patients (“Young”; 20–35 y) and (2) RA patients (“Aged”; 36–44 y), triggered with hCG. Cells were pooled, incubated for 24 h, and then collected and subjected to qPCR analysis of PEDF, VEGF, FSHR, and AMH expression. Number of pools in each experimental group: n > 10 (two-to-three women per pool; each black circle represents one pool). Genes were calibrated with HPRT-1. Data are presented as mean ± SEM. Results were analyzed by Student’s *t*-test with Welch’s correction, * pv ˂ 0.05.

Considering the detected effect of PEDF on mice oocytes’ PBE and the elevated mRNA level shown in the GCs of RA mice, we explored its involvement in human ovarian aging. We used the pooled hpGCs of young and RA IVF patients, triggered with hCG, and followed the expression of PEDF mRNA, as well as transcripts of VEGF, FSHR, and AMH mRNAs that are known to be involved in the maturation of the follicle. We observed higher levels of PEDF and VEGF mRNAs (approximately 2.0-fold change) in the hpGCs of the RA patients compared to the young patients (Figure 2); FSHR and AMH expression was not different between the GCs of young and RA patients.

### 2.3. Expression of PEDF Protein in Human Primary Granulosa Cells (hpGCs) and Follicular Fluid (FF)

The additional set of patients triggered with hCG were divided by age into two experimental groups, young and RA, and assessed individually for several parameters. The number of retrieved oocytes was not significantly different between the young and RA patients (11.5 ± 6.49 and 7.5 ± 5.28, respectively).

**Figure 3:**
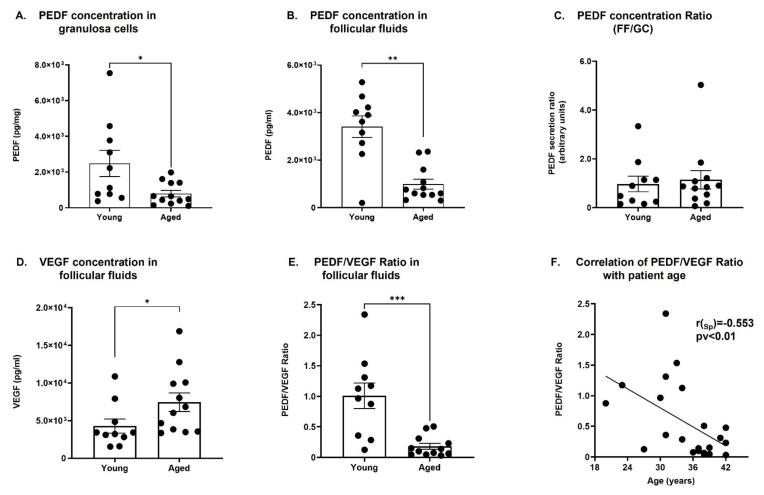
PEDF and VEGF protein in human GCs and in FF. phGCs extracts and FFs of IVF patients: phGCs and FFs of young (“Young”; 20–35 y; N = 10) and RA patients (“Aged”; 36–44 y; N = 11) were collected individually and subjected to specific ELISA assay kits. (**A**,**B**) The level of PEDF in GCs extracts (**A**) and in their corresponding FFs (**B**). (**C**) PEDF concentration ratio, calculated by dividing the level of PEDF in FFs by its level in GCs. (**D**) The level of VEGF in FFs. (**E**) PEDF/VEGF ratio in FFs. Each black circle represents one patient. Data are presented as mean ± SEM. Results were analyzed by Student’s *t*-test with Welch’s correction. * pv < 0.05; ** pv < 0.01; *** pv < 0.001. (**F**) Spearman correlation analysis of PEDF/VEGF ratio to patients’ age.

To follow the expression of PEDF protein level, we examined its concentration within the GCs and in their corresponding FFs, which constitutes the secreted portion of the protein. We found that the levels of both intra-cellular and secreted PEDF were decreased in RA patients compared to the levels in young patients (Figure 3A,B, respectively). The ratio between the secreted and intra-cellular PEDF protein level (FF/GCs) showed no significant difference between the age groups (Figure 3C), reflecting a similar secretion of the protein. In contrast, we detected a significant increase in the VEGF protein level in the FF of RA patients compared to the level in young patients (Figure 3D). The calculated PEDF/VEGF ratio in the FF of RA patients was decreased, and negatively correlated to the patients’ age (Figure 3E,F, respectively).

### 2.4. In Vitro Maturation of Mice Oocytes

**Figure 4:**
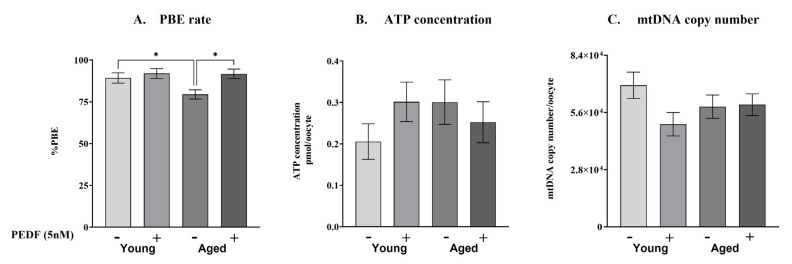
PBE rate, ATP concentration, and mtDNA copy number in mouse oocytes matured in vitro. GV oocytes were isolated from individual young (“Young”; 6–8 weeks old) and RA (“Aged”; 40–46 weeks old) ICR female mice, primed for 48 h with PMSG (5IU or 7.5 IU, respectively) and subjected to IVM with (+) or without (−) 5 nM rPEDF. (**A**) After 20 h of IVM, the PBE rate was calculated and recorded for each mouse individually. Total number of oocytes in each group: n ≥ 84. (**B**,**C**) MII oocytes (after IVM) were individually collected, lysed, and subjected to analysis of ATP concentration or mtDNA copy number. Total number of oocytes per experimental group: n ≥ 19. Each group contained oocytes from at least four mice. Data are presented as mean ± SEM. Results were analyzed by one-way ANOVA with FDR correction, * pv < 0.05.

To evaluate the role of PEDF in oocyte maturation, we followed in vitro maturation (IVM) in single oocytes of young and RA mice, with or without the supplementation of rPEDF to the culture medium (Figure 4). We found a significant decrease in the average rate of PBE in the oocytes of RA mice compared to the oocytes of young mice (79% and 89%, respectively; Figure 4A). rPEDF supplementation improved the PBE rate in the oocytes of RA mice towards reaching the value of young mice. When examining the ATP concentration and mtDNA copy number of oocytes that had completed their maturation (reached the metaphase II; MII stage; Figure 4B,C, respectively), we did not detect a significant difference in the measured energetic parameters; moreover, rPEDF supplementation did not affect the ATP concentration or mtDNA copy number of these oocytes.

## 3. Discussion

Reproductive aging is an emerging topic amongst the scientific community of fertility-related physicians and researchers due to the increasing age of childbearing [25]. The reproductive aging process, and, specifically, its onset, varies among patients. Therefore, the common use of age as a cutoff between groups led to variability of the measured values within the experimental group. Nevertheless, we could detect significant changes regarding PEDF, thus pointing to its importance even more.

For the last several years, our research focused on the role of PEDF in the female reproductive system. We have characterized its expression and demonstrated its importance in maintaining the physiologic ovarian activity [19,26,27]. Our latest study points at PEDF’s ability to alleviate oxidative damage in oocytes [21].

Several other studies have shown that PEDF expression changes with age [28,29], and suggested that it plays a key role in the development of angiogenic-related pathologies [30,31]. A reduction of PEDF mRNA and protein expression was reported in aged skin tissue, which contributes to age-associated cutaneous vascular dysplasia [32]. Conversely, an increase in PEDF mRNA and protein levels was detected in the mature kidneys of aging rats, probably due to an impairment of angiogenesis, which occurs with aging [33]. Another report that focused on PEDF as a therapeutic agent found that its administration prolonged the lifespan of mesenchymal cells by reducing OS and preserving their differentiation potential [34]. However, very few data were published regarding the involvement of PEDF in reproductive aging.

To explore the possible involvement of PEDF in reproductive aging, we used female mice (40–46 weeks old) that presented the initial stages of ovarian aging (average litter size at weaning was 10.5 compared to 12.7 in young females). These mice were still fertile, but had a smaller litter size, equivalent to ~40-year-old women [35]. In a recent study [36], the researchers used ICR mice as a model for examining the effect of α-ketoglutarate in relation to age. They reported that at 8 months of age, the litter size declined significantly compared to young, 8-week-old mice (average litter size: ~12 and ~14, respectively). We demonstrated a significant increase in the expression of PEDF mRNA in both human and mice pGCs models. This finding, which is at odds with the data presented by Ye et al. [15], could be explained by the difference in the examined follicles’ developmental stage (GCs in our experiments were retrieved from large, fully-grown follicles) and in the age of the mice that were used.

We further followed the expression of PEDF in RA women, as well as the mRNA levels of VEGF, FSHR, and AMH. As in the model of RA mice, we found a significantly elevated level of PEDF in the pGCs of RA patients. We also detected an increase in VEGF mRNA level. Moreover, in the pGCs of both RA mice and humans, we did not find changes in the expression of AMH mRNA compared to young females. One of the indicators of the reproductive potential of RA women is decreased AMH level in the serum, but it was found [37] that its mRNA level in mural GCs, the equivalent cells to hpGCs used by us, was not changed in relation to age. Our finding supports that data, even-though we examined a younger cohort of women and used hpGCs isolated from pooled follicles aspirated from the same woman, and not from one leading preovulatory follicle only, as reported by Kedem et al. [37]. There is only scarce information regarding changes in the level of FSHR mRNA during aging. We found no change between the age groups; thus, the clinically poor response of RA females to FSH may be attributed to faulty signal transduction within the cells [38].

Next, to examine the expression of PEDF protein, we used hpGCs and FFs of individual young and RA patients, triggered with hCG. The average number of oocytes retrieved from these patients did not differ between the age groups. We followed the levels of PEDF protein in the GCs and in their corresponding FFs. We found that the intra-cellular concentration of PEDF protein was significantly lower in RA patients than in young patients. Our former observation of the elevated PEDF mRNA level in the GCs of RA patients, concomitant with a decrease in its intra-cellular protein level, could point at flaws in the translational mechanisms, or changes in the stability of the protein produced by the hpGCs of RA patients. During aging, a general reduction in protein synthesis is observed [39]. The transformed transcriptome of somatic-aged cells of many tissues is attributed to faulty transcription, translation, and protein control within the cell [40]. For example, under stress, as during cellular aging, the integrated stress response (ISR), an intra-cellular signaling network that maintains the protein balance in response to variable environmental changes, is highly activated, i.e., it reduces global mRNA translation while enhancing the expression of stress response genes [39,41,42]. This results in discordance between mRNA and protein levels. Similarly, in various species, RA oocytes experience a decline in their ability to carry out mRNA translation [43]. The elevation in PEDF mRNA in RA GCs could derive from excessive transcription, as feedback to the decline in protein level.

We also found a decrease in the level of PEDF protein in the FF; however, the FF/GC ratio was similar in both age groups, implying that there is no change in the secretion of the protein into the FF. Our finding of an elevated concentration of VEGF protein in the FF of RA patients, in accordance to the literature [44,45], point to a gene-specific mechanism and not a general property of aging. VEGF elevation, along with PEDF reduction, intensified the inverted relationship between PEDF and VEGF, as demonstrated in various models [30,46,47]. Consequently, the calculated PEDF/VEGF protein ratio is significantly decreased in RA patients, and inversely correlated with patients’ age, suggesting an impaired angiogenic balance at the onset of ovarian aging. We suggest that the measurement of PEDF protein level could be an informative and reliable method for detecting the initiation of reproductive aging in women.

We have previously demonstrated the ability of rPEDF to overcome the inflicted damage caused by H_2_O_2_ [20,21]. Because aging is characterized by elevated OS, we tested the therapeutic potential of rPEDF on the oocytes from the ovaries of RA mice by challenging the oocytes of young and RA mice to undergo IVM, and determined their PBE rate. The average PBE rate in the oocytes of RA mice was lower than in the oocytes of young mice. This relatively small, though significant, decrease points at the very onset of the reproductive aging process. rPEDF supplementation has improved the hampered maturation (PBE) rate of the oocytes from RA mice, expressed by more energetically competent oocytes that reached the MII stage.

## 4. Materials and Methods

### 4.1. Animals

Young ICR (an outbred line originated from the Institute of Cancer Research in the USA) female mice (“Young”; 6–8 weeks old) and RA mice (“Aged”; 40–46 weeks old that were retired from the breeding colony) were purchased (Envigo Ltd., Jerusalem, Israel; ISO9001:2015) and housed in the air-conditioned, light-controlled animal facilities of the Sackler Faculty of Medicine in Tel-Aviv University. Animal care was in accordance with institutional guidelines, and was approved by the Institutional Animal Care and Use Committee (permit no., 01-18-053).

### 4.2. Oocyte Collection and Culture

Young and RA mice were subcutaneously administered with pregnant mare serum gonadotropin (PMSG; Syncro-part, Sanofi, Paris, France; 5 IU and 7.5 IU, respectively). Forty-eight hours later, GV oocytes from each mouse were collected, as previously published [21]. Briefly, using a 21G needle, whole ovaries were gently punctured, and GV oocytes were released into pre-warmed (37 °C) L-15 culture medium (Biological Industries, Beit-Haemek, Israel), supplemented with penicillin (100 IU/mL); streptomycin (100 mg/mL) (Biological Industries); and milrinone (1 μM; M-4659; Sigma-Aldrich, St. Louis, MO, USA), a phosphodiesterase III inhibitor that prevents spontaneous resumption of the first meiotic division, keeping the oocytes at the GV stage. The oocytes were mechanically stripped of their surrounding cumulus cells; washed in αMEM medium (Thermo Fisher Scientific, Waltham, MA, USA) supplemented with penicillin (100 IU/mL), streptomycin (100 mg/mL) (Biological industries), and 10% FBS (maturation medium); and randomly divided into 2 groups to be cultured either with or without 5nM rPEDF (ab56289, Abcam, Cambridge, UK). The oocytes were further incubated in vitro for 20 h to allow spontaneous maturation (IVM), as previously described [21], and reach the MII stage (indicated by the presence of a PB). We followed the rate of PBE: on each experimental day, the PBE rate was calculated individually for each mouse in both treatment groups. Only oocytes that were able to reach MII were individually collected for ATP and mtDNA copy number analysis (as elaborated below).

### 4.3. ATP Detection

MII oocytes were washed in nuclease-free water and transferred individually into a tube in a total volume of 10 µL and stored at −20 °C until ATP testing. The concentration of ATP in the oocytes was determined as previously described [21], using a specific luminescence kit according to the manufacturer’s protocol (StayBrite™ Highly Stable ATP Bioluminescence Assay Kit, BioVision, K791-100; Milpitas, CA, USA).

### 4.4. Quantification of mtDNA Copy Number

MII oocytes were transferred individually into 10 µL lysis buffer (50 mM Tris-HCl (T1503), pH 8.5; 0.1 mM EDTA (E-0399); 0.5% Tween-20 (P1379); 200 μg/mL proteinase K (124568, Sigma-Aldrich)) and lysed as previously described [48]. The absolute number of mtDNA copies in each oocyte was determined by qPCR, using specific primers (F5′TACCTCACCATCTCTTGCTA3′; R5′GCTACACCTTGACCTAACG3′) that detect a specific mtDNA sequence [12]. This sequence (114 bp) corresponds to the mitochondrial 12s ribosomal RNA subunit coding region of the mtDNA sequence (mt-Rnr-1). An accurate number of mtDNA copies in a single oocyte was calculated as previously described [21] by generating a standard of synthetic puc57-Amp plasmid inserted with the 114 bp mtDNA fragment (GENEWIZ; Chelmsford, MA, USA), containing corresponding sequences for the above mtDNA primers. A calibration plot of the known copy numbers of the plasmid and the corresponding ΔCT was used to determine the absolute copy number of mtDNA in each oocyte.

### 4.5. Mice Primary Granulosa Cells (mpGCs)

Cells were isolated as previously described [46,49], with some modifications, as elaborated. Follicles were punctured with a 21G needle, and the GCs were released out of the large antral follicles into the culture medium, which was then filtered through a cell strainer (0.4 µM, Corning, Phoenix, AZ, USA) to avoid debris contamination. The filtered GCs were centrifuged (300× *g* for 5 min) and seeded into 6-well plates (Nunc, Denmark) in a pre-heated DMEM-F12 medium supplemented with 10% FBS, 2 mM L-glutamine, penicillin (10,000 IU/mL), and streptomycin (10 mg/mL) (Biological industries) for 24 h in a humidified incubator at 37 °C and 5% CO_2_ in the air. The seeding of GCs was performed individually for each mouse. The confluence of cells was around 70% in each well. After the incubation, the cells were harvested for RNA analysis.

### 4.6. Human Primary Granulosa Cells (hpGCs) and Follicular Fluid (FF)

Women, 20–44 years old, undergoing in vitro fertilization (IVF), participated in this study after providing written informed consent forms (IRB approval 0240-19, Shaare Zedek Medical Center, Jerusalem, Israel). To promote oocyte maturation, the patients received hCG (Ovitrelle; 250 mg; Merck Serono) 36 h prior to ovum pick up (OPU). We have previously shown [46] that hCG- or GnRH-a-triggering differentially regulate the PEDF expression in hpGCs. As RA patients are triggered with hCG rather than GnRH-a, we decided to include young and RA patients, triggered with hCG in our experiments. The hpGCs were isolated from aspirated follicular fluids (FF) as previously described [46], and cultured in DMEM-F12 (Biological industries) medium supplemented with 10% FBS, 2 mM L-glutamine, penicillin (10,000 IU/mL), and streptomycin (10 mg/mL) to allow cell adherence to the dish and ensure the collection of hpGCs only.

The cells designated for RNA analysis were cultured for 24 h to allow the maximal adherence of cells to a culture dish. The cells were seeded in 60–70% confluence with DMEM-F12 medium supplemented with 1% FBS, 2 mM L-glutamine, penicillin (10,000 IU/mL), and streptomycin (10 mg/mL) (Biological industries). To reach the desired cell confluence in a culture dish, the seeding was performed in pools (2–3 women per pool; N = pool) and sorted by age into two groups: (I) young patients (“Young”; ≤35 y, average age = 29.9 ± 3.9 y; N = 11 from 23 patients) and (II) RA patients (“Aged”; ≥36 y, average age = 40.2 ± 2.4 y; N = 10 from 16 patients), triggered with hCG. Cells were lysed with Trizol reagent (Bio-Tri; Bio-lab, Jerusalem, Israel) for further RNA purification.

The analysis of protein level was performed in samples of FFs and their corresponding GCs, aspirated from individual young patients (“Young”; ≤35 y, average age = 29.4 ± 4.7 y; N = 10) and RA patients (“Aged”; ≥36 y, average age = 39.1 ± 2.1 y; N = 11), triggered with hCG. The cells from each woman were seeded separately (60–70% confluence; yet, not in pools) and incubated as described above. Afterwards, cells were lysed as described in [19]. The protein supernatant of the GCs and clear FFs samples (after additional centrifugation at 2000× *g* for 15 min) were stored at −80 °C until the ELISA assay.

### 4.7. RNA qPCR

Total RNA was extracted from human (pooled) or mouse GCs using Trizol reagent (Bio-Tri; Bio-lab, Jerusalem, Israel) according to the manufacturer’s instructions, and quantified with the Nano-Drop spectrophotometer (ND-1000; Thermo Scientific). The total RNA was reverse-transcribed using a high-capacity cDNA Reverse transcription kit (Applied Biosystems (ABI), Foster City, CA, USA) according to the manufacturer’s instructions. Changes in the level of expression of mRNA were detected by real-time PCR (qPCR), performed using SYBR green reagent (Power Fast SYBR Green PCR Master Mix; Applied Biosystems) or TaqMan Fast reagent (TaqMan Fast Advanced Master Mix; Applied Biosystems), along with 15 ng cDNA and specific primers, on a StepOnePlus Real-Time PCR System (Applied Biosystems). The relative gene expression (comparative ΔCt) was evaluated using the specific primers, listed below, and normalized to the expression of HPRT (human) or to RPLP2 (mouse), which served as endogenous controls. The stability of reference genes expression was verified among all samples and experiments.

(1) Taqman probes: PEDF, Hs01106937_m1; VEGF, Hs00900055_m1; AMH, Hs00174915_m1; AMHR, Hs01086646_g1, PEDF, Mm00441270_m1; VEGF, Mm00437306_m1; AMH, Mm004310795_g1; FSHR, Mm00442819_m1; RPLP2, Mm00782638_s1.
(2) Specifically designed primers: human FSHR (F5′GGTGCATTTTCAGGATTTGG3′; R5′CTGCCTCTATCACCTCCAAGA3′),

Human HPRT (F5′TGACACTGGCAAAACAATGCA3′; R 5′CTGCCTCTATCACCTCCAAGA3′).

### 4.8. Enzyme Immunoassay

The level of PEDF in GCs lysates, and the levels of both PEDF and VEGF in FFs of IVF patients were determined using specific ELISA kits (ab24653, ab-222510, respectively; Abcam) according to the manufacturer’s protocol.

### 4.9. Statistics

All statistical analyses were performed by the GraphPad Prism 9.0.0. software (GraphPad Software, Inc., San Diego, CA, USA). The PBE rate, ATP concentration, mtDNA copy number, levels of protein, and mRNA in the FF are expressed as standard error of the mean, and are compared as appropriate for data distribution. Data normality was assessed using Kolmogorov–Smirnov tests. The data were then evaluated by either Student’s *t*-test to specify the significance between two experimental groups, followed by Welch’s correction if the groups’ variance was not equal, or by ANOVA to specify significance between more than two experimental groups, followed by FDR correction for multiple comparisons. A *p*-value < 0.05 was considered statistically significant.

## 5. Conclusions

The ovarian aging process is vastly studied, mainly because of the global trend of postponing childbearing age. Few ovarian reserve markers that predict the onset of ovarian aging were published: antral follicle count, basal FSH, and AMH levels. Our study following the late stages of folliculogenesis, i.e., oocyte maturation, GCs transition during ovulation, and FF production, points to the involvement of PEDF in the early onset of reproductive aging, and leads us to propose that PEDF can serve as an early marker of this process. Moreover, based on our findings of low PEDF protein levels, which may possibly point to flaws in the process of PEDF translation within the GCs of RA patients, PEDF can be considered as a therapeutic supplement candidate in ART. However, the mechanism of this process is to be determined by further research.

## 6. Declarations

Ethics approval and consent to participate: Mice—animal care was in accordance with institutional guidelines, and was approved by the Institutional Animal Care and Use Committee (permit no., 01-18-053). Humans—patients participated in this study after providing written informed consent forms (IRB approval 0240-19, Shaare Zedek Medical Center, Jerusalem, Israel).

## Data Availability

All data generated through this study are included in this article.
